# Prevalence of eye pathology in a group of diabetic patients at National District Hospital Outpatient Department in Bloemfontein, South Africa

**DOI:** 10.4102/phcfm.v9i1.1440

**Published:** 2017-09-27

**Authors:** Joleen P. Cairncross, Wilhelm J. Steinberg, Mathys J. Labuschagne

**Affiliations:** 1 Department of Family Medicine, Faculty of Health Sciences, University of the Free State, South Africa; 2 Clinical Simulation and Skills Unit, Faculty of Health Sciences, University of the Free State, South Africa

## Abstract

**Background:**

Diabetic retinopathy is the third most common cause of blindness after cataracts and glaucoma in South Africa. Primary healthcare interventions providing eye care services play an important role in preventing complications.

**Aim:**

To determine the prevalence of eye pathology in a group of diabetic patients at National District Hospital by screening for diabetes-associated ocular pathology.

**Setting:**

Outpatients Department run by Department of Family Medicine at National District Hospital in Bloemfontein from June to July 2014.

**Methods:**

Interviews were used to collect information regarding diabetic patients’ history of diabetes mellitus and if and when previous diabetic retinopathy screening was performed. Visual acuity was assessed, intra-ocular pressure measured and a non-mydriatic digital fundus camera used to screen for retinal pathology.

**Results:**

During the last year, only 4.5% of patients had their vision checked with a Snellen chart, and 16.5% were examined with an ophthalmoscope. Since diagnosis of diabetes, only 15.5% of patients were referred to an ophthalmologist. Patient referral was needed for 87 (42.9%) cases for refractive disorders, 37 (18.2%) for suspected glaucoma, 30 (14.8%) for cataracts, and 22 (10.8%) for diabetic retinopathy.

**Conclusion:**

This study confirms that glaucoma, cataracts and diabetic retinopathy are prevalent eye conditions among diabetic patients. Offering eye screening at primary healthcare level may contribute to early detection of eye pathology and timeous referral for sight-saving treatment.

## Introduction

In 2010, the World Health Organization (WHO) estimated that 285 million people globally are visually impaired, of which 90% are living in low-income settings.^1,2^ In 1999, the WHO launched Vision 2020, a global plan for the elimination of avoidable blindness by 2020.^3^ The South African National Prevention of Blindness Programme^4^ is a signatory of this global initiative. The leading cause of blindness in South Africa is cataracts.^4^ Other causes are uncorrected refractive error, glaucoma, diabetic retinopathy and age-related macular degeneration.^3,4^ The current magnitude of visual disability in South Africa, which is projected to increase exponentially over the coming decades, has far-reaching social, economic and quality of life implications for the affected individuals, their families and communities.^4^ From the 2011 South African census on visual impairment, the Free State Province had the highest prevalence at 13.8%, followed by the Northern Cape (11.5%) and North West (11.3%).^5^

It is estimated that 415 million people globally suffer from diabetes, and of those 14 million people are from the African region.^6^ In 2015, 2.28 million cases of diabetes were reported in South African adults between the ages of 20 years and 79 years.^6^ Nonetheless, South Africa has not prioritised the targets set out by the Global Diabetes Scorecard of the International Diabetes Foundation and remains more focused on tuberculosis and HIV/AIDS. Furthermore, there is still no official framework for the surveillance of diabetes, and comprehensive services for diabetes care and prevention are not implemented universally.^7^

The WHO aims to stimulate and support the adoption of effective measures for the surveillance, prevention and control of diabetes and its complications, particularly in low- and middle-income countries. Screening for and treatment of retinopathy are considered effective cost-saving interventions that would reduce morbidity related to diabetes and especially diabetes-related blindness. Sighted individuals can retain their independence and reduce the financial and social burden on the state and society.^8,9^ The strategy for the prevention of avoidable blindness and visual impairment is based on three core elements: strengthening disease control, human resource development, and infrastructure and technology. Primary healthcare and community-based interventions with the provision of high-quality eye care services play an important role to achieve these goals. A crucial shortage of eye care personnel persists in many low-income countries. Many countries in Africa have less than one ophthalmologist per million inhabitants.^10^

In South Africa, diabetic retinopathy is the third most common cause of blindness in diabetics after cataracts and glaucoma.^4^ The guidelines of the Ophthalmological Society of South Africa (OSSA) state that every diabetic person should have his or her eyes screened at least once a year for diabetic retinopathy, which should consist of a dilated retinal examination by an ophthalmologist. Alternatively, a dilated fundus photograph should be evaluated by an ophthalmologist, medical doctor, nurse or optometrist certified by OSSA.^11^ A pilot project screening for diabetic retinopathy in primary care at three Cape Town community healthcare centres assessed 400 patients with the aid of a mobile fundus camera. More than 80% had significantly reduced visual acuity and 63% had retinopathy (22% severe non-proliferative, 6% proliferative and 15% maculopathy). Referral for cataracts was necessary in 27% of cases, in 7% for laser treatment and in 4% for other specialist services.^12^

In a study by Germain et al.,^13^ it was confirmed that digital retinal photography screening by an ophthalmologist and a trained endocrinologist was equally reliable. Several studies confirmed that if non-ophthalmologists are trained in reading the fundus photographs, diabetic retinopathy can be accurately diagnosed.^9,14,15^ By making use of trained healthcare professionals to assist with the screening, the workload on ophthalmologists could be reduced. In the Free State, diabetic retinopathy screening is mainly performed at tertiary healthcare level at endocrinology and ophthalmology clinics. By introducing screening programmes for diabetic retinopathy on primary and secondary levels, the workload on tertiary ophthalmology institutions could be reduced to focus more on specialist care such as laser, cataract surgery and vitreo-retinal surgery. In a study by Khan et al.,^9^ it was confirmed that digital non-mydriatic fundus photography is an effective cost-effective screening and diagnostic tool for diabetic retinopathy in the South African primary care setting.

The aim of this study was to describe the prevalence of eye pathology in a group of diabetic patients attending the Outpatients Department supported by the Department of Family Medicine at National District Hospital (NDH), Bloemfontein, by assessing visual acuity, measuring intra-ocular pressure (IOP) and using non-mydriatic digital fundus photography as a screening tool. The objectives of this study were to: (1) estimate the prevalence of eye pathology, (2) determine if eye screening will improve early detection and referral to appropriate services and (3) determine whether diabetic patients had undergone a previous diabetic retinopathy screening.

## Research methods and design

### Study design and setting

A prospective descriptive study design was used. The study was conducted from June to July 2014 at a general Outpatients Department of NDH, Bloemfontein, South Africa.

### Study population and sampling strategy

Patients attending the Outpatients Department are referred from inpatient wards and the emergency room at NDH as well as by primary healthcare clinics in the Mangaung district. Diabetic patients are seen daily, and on average 180 to 220 outpatients are seen every month.

A convenience sample was selected from the routine diabetic follow-up patients. Eye care screening was added to the routine follow-up care. The researcher explained and offered this service to each diabetic patient. All patients presenting at the Outpatients Department during the study period who were 18 years or older with confirmed type 1 or type 2 diabetes mellitus and on medication were included. Patients who declined to participate were excluded. Two hundred and three patients were enrolled out of an estimated 240 diabetic patients who attended the Outpatients Department during the study period.

### Data collection

Information for this study was collected by means of an interview with the patient as well as collecting some of the information from the patient’s file. Information was recorded on a questionnaire and datasheet by the main author. Snellen visual acuity, IOP measurement and non-mydriatic digital fundus photography were used for the ophthalmic screening. A blood sample for HbA_1c_ level was taken if no results were available within the last 3 months.

A unique reference number was allocated to each patient. Fundus photography images were stored on the computer by using the reference number and date of birth of each patient. The reference number was linked to the patient’s name on a separate datasheet that was only available to the main author.

#### Patient interview

The researcher conducted a short interview with each patient to obtain demographic information, the duration of diabetes treatment, duration of attending the Outpatients Department, number of annual visits, as well as past diabetic retinopathy screening.

#### Eye examination

The main author, a family practitioner, was trained by an ophthalmologist in the correct execution of the examination techniques. Reliability was ensured by correlating the results with the ophthalmology registrar’s findings of the referred patients. Visual acuity of each patient was assessed with a Snellen chart, and pinhole visual acuity was used to screen for refractive errors. IOP was measured with an iCare^®^ tonometer. A direct ophthalmoscope was used to assess pupil reflexes and appearance of red reflex. The eclipse test was done to screen for a shallow anterior chamber to determine whether pharmacological dilation of a pupil can safely be performed without the risk of precipitating acute closed-angle glaucoma.

#### Non-mydriatic fundus photography

Screening for diabetic retinopathy was done by means of a Canon^®^ non-mydriatic digital fundus camera. In few cases where poor view of the retina was present, the main author administered one drop of 1% Tropicamide in both eyes to ensure dilation of pupils after the eclipse test had been performed. Fundus photographs were taken and images stored as JPEG files on the computer hard drive and a flash drive.

At weekly intervals, a senior registrar from the Department of Ophthalmology reviewed the datasheet and fundus photography images, and graded the diabetic retinopathy. In the event of photos that could not be graded, patients were referred to the ophthalmology clinic for full ophthalmological examination of the eyes.

The diabetic retinopathy grading scale of the OSSA was used to classify patients (Table 1).^16^ This grading system is based on the Scottish retinopathy grading system and recommendations.^17^

**TABLE 1:** Scottish diabetic retinopathy grading scheme and recommendations.^17^

Classification		Observation	Recommendations
R0	No diabetic retinopathy anywhere	No lesions	Re-screen 1 year
R1	Mild background diabetic retinopathy	At least one dot haemorrhage or micro aneurysm, with or without hard exudates	Re-screen 1 year
R2	Observable background diabetic retinopathy	Four or more blot haemorrhages, in one hemi-field only	Re-screen 6 months
R3	Referable background diabetic retinopathy	Four or more blot haemorrhages, in both inferior and superior hemi-fields, venous beading, IRMA (Intraretinal Microvascular Abnormality)	Refer ophthalmologist (may need laser)
R4	Proliferative diabetic retinopathy	Active new vessels, vitreous haemorrhages	Refer ophthalmologist (may need laser)
R6	Inadequate	Not adequately visualised	Dilate & re-screen. Refer if R3/R4/M1/M2
M0	No maculopathy	No lesions	Re-screen 1 year
M1	Observable maculopathy	Lesions in a radius of > 1 but < 2 disc diameters of centre of fovea. Any hard exudates	Refer ophthalmologist
M2	Referable maculopathy	Lesions in a radius of < 1 disc diameter of centre of fovea. Any blot haemorrhages. Any hard exudates	Refer ophthalmologist

*Source*: Scottish Diabetic Retinopathy Screening Collaborative 2003^17^

In this study, the referral guidelines of the International Council of Ophthalmology guidelines for diabetic eye care are adopted and used to refer patients to the Free State Department of Ophthalmology^18^: The guidelines are:

- Visual acuity normal and no signs of diabetic retinopathy (background or pre-proliferative or proliferative changes) should be referred within 3 months.
- Visual acuity reduced and no signs of diabetic retinopathy (background or pre-proliferative or proliferative changes) should be referred within 1 month.
- Possible maculopathy, irrespective of visual acuity and with signs of diabetic retinopathy (background or pre-proliferative changes) should be seen within 1 month by an ophthalmologist.
- Irrespective of visual acuity and with signs of proliferative diabetic retinopathy should be seen immediately by an ophthalmologist.

### Data analysis

The analysis was performed by the Department of Biostatistics, Faculty of Health Sciences at the University of the Free State (UFS). Descriptive data are reported using frequencies, percentages, medians, means and standard deviations (SD).

### Ethical considerations

The study was approved by the Research Ethics Committee of the Faculty of Health Sciences, UFS (79/2014). Permission to conduct the study was obtained from the Head, Free State Department of Health, the consultant from the Department of Family Medicine in charge of the Outpatients Department at NDH and the Heads of the Departments of Ophthalmology and Optometry at UFS. Patient written consent was required for participation.

## Results

Of the 240 patients with diabetes who attended the Outpatients Department at NDH during the study period, 203 patients were included in the study. In all, 200 questionnaires were analysed, as three questionnaires were lost during the study.

The majority of the participating patients were female (72.5%). The home language of 60.0% of the patients was SeSotho, while 17.0% were Afrikaans speaking. The mean age of the patients was 57 years (range 30 years – 88 years). One patient had a prosthetic left eye.

### Diabetes medical history

The mean diabetes treatment duration for the patients was 8.4 years (0.1 years – 40.0 years). Patients had been attending the Outpatients Department for a mean of 5.2 years (0.1 years – 30.0 years) and the mean number of routine visits per year was 3.4 (0 visits – 20 visits).

### Past diabetic retinopathy screening

In response to questions relating to eye screening during their routine visits over the last year, 23.0% of the patients mentioned to their doctor that their vision was deteriorating, while 12.0% indicated that the doctor enquired about their vision. Only 3.5% indicated that their vision was checked with a Snellen chart and 16.5% had their eyes examined with a direct ophthalmoscope within the last year.

The majority of the 200 patients (68.0%, *n* = 136) were aware that diabetes could adversely affect their vision, while 27.5% (*n* = 55) were not sure. Only 15.5% (*n* = 31) of the patients screened had ever been referred to an ophthalmologist since the diagnosis of diabetes.

### Eye examination

#### Visual acuity

The visual acuities of the participants are summarised in Table 2. The participants with a visual acuity of 6/12 and 6/9 improved with spectacles/pinhole, indicating refractive errors. Normal to mild visual impairment was found in 83.0% of right eyes and 83.2% of left eyes.

**TABLE 2:** Visual acuity of patients.

Visual acuity†	Without spectacles/pinhole		With spectacles/pinhole	
	Right eyes (%) *n* = 203	Left eyes (%) *n* = 202	Right eyes (%) *n* = 203	Left eyes (%) *n* = 202
No light perception	1.0	1.5	1.0	1.5
Light perception	0.0	0.5	0.0	0.5
Finger counting	4.9	3.9	4.4	3.0
6/60	7.4	6.9	1.0	2.0
6/36	8.9	9.4	3.0	3.5
6/24	13.8	6.4	5.9	4.4
6/18	20.2	27.6	19.2	21.2
6/12	19.7	19.2	26.6	24.6
6/9	20.2	17.7	33.0	31.5
6/6	2.0	4.9	3.9	5.9
No visual acuity tested	2.0	2.0	2.0	2.0

*Source*: South African National Prevention of Blindness 2002.^4^

† Visual acuity in better eye with best correction (with a Snellen chart): 6/6–6/18 (normal); < 6/18–6/60 (visual impairment); < 6/60–3/60 (severe visual impairment); < 3/60 (blind).

#### Pupil reflexes, appearance of red reflex and eclipse test

Examinations performed with a direct ophthalmoscope showed a relative afferent pupillary defect present in one (0.5%) of the right eyes and two (1.0%) of the left eyes. The red reflex was present (includes ‘present’, and ‘present but reduced’) in 97.0% of both eyes. The eclipse test was negative in 98.5% (*n* = 200) of right eyes and 100% (*n* = 202) of left eyes indicating that almost all participants had open angles.

#### Intra-ocular pressure

In one patient, it was difficult to perform the IOP in both eyes; therefore, only the right eye was tested. Results for the right eyes (*n* = 202) showed a mean IOP of 17.3 mmHg (median 17.0 mmHg, SD 6.4) compared to 17.1 mmHg (median 16.0 mmHg, SD 5.9) for the left eyes (*n* = 200). Measurements ranged from 8.0 to 68.0 mmHg for the right eyes and 8.0 to 60.0 mmHg for the left eyes. The maximum IOP results came from one patient.

#### Non-mydriatic fundus photography

Only one patient’s pupils needed pharmacological dilation for a better view. Poor image quality (inadequate view) was because of cataracts, vitreous haemorrhages, technical difficulty in operating the equipment and poor patient cooperation (Table 3).

**TABLE 3:** Grading of the images obtained with a non-mydriatic fundus camera.

Variables	Right eyes (*n* = 203)		Left eyes (*n* = 202)	
	*n*	(%)	*n*	(%)
**Fundus**				
Gradable images†	185	91.1	179	88.1
No diabetic retinopathy	145	78.4	146	81.6
Mild diabetic retinopathy	19	10.3	18	10.1
Observable background diabetic retinopathy	8	4.3	5	2.8
Referable background diabetic retinopathy	5	2.7	4	2.2
Proliferative diabetic retinopathy	5	2.7	6	3.4
Inadequate view	15	7.4	20	9.9
No photo	3	1.5	3	1.5
**Macula**				
Gradable images	190	93.6	187	92.1
No maculopathy	165	86.8	164	87.7
Observable maculopathy	12	6.3	13	7.0
Referable maculopathy	13	6.8	10	5.3
Inadequate view	9	4.4	12	5.9
No photo	4	2.0	3	1.5

† Fundus images were graded according to the Scottish Diabetic Retinopathy Grading Scheme and Recommendations^17^ (Table 1).

In 146 of the patients, no diabetic retinopathy was noted, 19 of the patients had mild diabetic retinopathy, 5 patients presented with referable background diabetic retinopathy and 5 with proliferative diabetic retinopathy. These 10 patients were referred immediately to the Department of Ophthalmology for the appropriate treatment. Fifteen patients presented with referable maculopathy and 19 with observable maculopathy. Some of the ocular pathologies were not present in both eyes, as indicated in Table 3.

Other ocular pathologies identified during the study included refractive errors (*n* = 86, 43.2%), cataracts (*n* = 56, 27.6%), glaucoma suspects (*n* = 37, 18.2%) and hypertensive retinopathy (*n* = 24, 11.8%). The other ocular pathologies (*n* = 49, 24.1%) included blindness, age-related macular degeneration, suspected meningioma, macular hole, macular scar, pterygium, pinguecula, strabismus, chorioretinal disorders, disorders of vitreous body, other disorders of lens, optic nerve and visual pathway disorders, and asteroid hyalosis.

### Diabetic control measure (blood HbA_1c_ levels)

From the 203 patients in this study, 195 results of blood HbA_1c_ levels were found in their files. The mean HbA_1c_ level for the patients was 8.4% (range 5.4% – 13.1%; SD 2.0) with a median of 8.0%. Almost 70% (*n* = 134; 68.7%) of the patients had HbA_1c_ levels higher than 7%, where a HbA_1c_ level < 7% is considered reasonable.^15^

### Patient referral

Of the 203 patients screened, 42.9% (*n* = 87) were referred to the Department of Optometry for a refractive disorder and 47.8% (*n* = 97) to the Department of Ophthalmology for other ocular pathology. A patient could have been referred for more than one ocular disease.

Reasons for referral to the Department of Ophthalmology included glaucoma suspects (*n* = 37; 18.2%), cataracts (*n* = 30; 14.8%) diabetic retinopathy (*n* = 22; 10.8%) and various other ocular diseases (*n* = 23; 11.3%) as mentioned earlier.

## Discussion

Findings of this study are in line with the three main causes of blindness in South Africa, which are refractive disorders, cataracts and glaucoma.^4^ Preventing and treating visual impairment have a significant impact on the quality of these patients’ lives – socially and economically. Furthermore, diabetic patients have a higher risk for the development of glaucoma^19^ and cataracts,^15^ impacting even more the vision of the diabetic patient.

The WHO^3^ and the South African National Prevention of Blindness Programme^4^ aim to eliminate avoidable causes of blindness by 2020 using primary healthcare and community-based interventions. This is the first study done at primary healthcare level on diabetic retinopathy screening performed in Bloemfontein. The study clearly demonstrates that the Outpatient Department is falling short of the current recommended annual diabetic retinopathy screening protocol as recommended by SEMDSA and OSSA.^15,16^ This is in line with a study performed in Rwanda at health centres in one district where participants reported that the primary care providers did not communicate information about eye care and failed to perform basic eye screening.^20^ This highlights the need to educate both the general public and healthcare professionals about eye diseases and conditions such as glaucoma, diabetic eye disease, age-related macular degeneration and low vision.^21^ The majority of patients in the study were aware that diabetes could adversely affect their vision; however, only 15.5% of patients had ever been referred for diabetic retinopathy screening since they were diagnosed. This suggests that patients had not been educated that annual eye examination is required in all diabetics. The health providers also do not seem to practise this guideline. Patients often arrive at the ophthalmology clinic with significant visual impairment that could have been prevented or mitigated, had it been diagnosed earlier.

Diabetic retinopathy screening that is not done by an ophthalmologist, usually comprise visual acuity screening and retinal examination.^15^ In this study, 42.9% of the patients required referral because of refractive disorders. Where visual acuity does not improve with spectacles or pinhole, the underlying pathology is less likely a refractive disorder.^22^ In this study, approximately a third of the eyes of patients examined had significant visual impairment that could affect the ability to obtain/retain a driver’s licence for a standard motor vehicle.^23^ In a similar study in Cape Town at primary healthcare level (*n* = 400), reduced visual acuity and reduced red reflex were observed in 84% of patients.^12^

With the use of a direct ophthalmoscope, an opacification of the lens (suggestive of a cataract) was easily identified in almost a third (27.6%) of patients. Just over half of those patients needed referral to an ophthalmologist for cataract surgery. A study done in the Mopani district found that cataracts were more prevalent in left eyes of male (52.8%) and right eyes (39.3%) of female patients with visual impairment.^24^ Patients with cataracts in the Cape Town study accounted for 35% of the cases in the study.^12^ Almost all of the patients in this study had a negative eclipse test indicating that the patients did not have occludable angles, and dilation of pupils could safely be done in most patients at primary healthcare level or where diabetic retinopathy screening is performed.

Screening for raised IOP (above 20 mmHg), an indication of possible glaucoma, was performed with an iCare^®^ tonometer with the results available immediately. As diabetic patients have a higher risk for glaucoma, it is useful to add this to the eye screening of patients.^19^ After refractive disorder, glaucoma/glaucoma suspects was the second most prevalent eye disease (18.2%) identified in this study. Glaucoma prevalence in the Mopani district was much lower at 3.6%.^24^ In our study, an iCare^®^ tonometer, fundus photography and the assistance of an ophthalmology registrar were used to diagnose glaucoma suspects, possibly resulting in a higher accuracy than in the Mopani district.

Screening for retinopathy is currently lacking in diabetes care at a primary care level in many settings in South Africa. A study by Khan et al.^9^ showed that non-mydriatic fundus photography is a cost-effective method to reduce diabetes-related blindness. In our study, a Canon^®^ non-mydriatic fundus camera was used as a screening tool that contributed to a more objective and reliable fundus screening than dilated fundus examination with a direct ophthalmoscope alone. The camera is also easy to use by any healthcare worker, with good patient cooperation. In a pilot study in South Africa, 400 patients were screened using a non-mydriatic mobile fundus camera. The study showed that screening with a camera is feasible in the primary healthcare sector, and noted that one technician should be able to photograph almost 10 000 patients yearly.^9,12^ The use of a mobile fundus camera has also been described as an innovative approach to reduce preventable blindness on a national level in South Africa. It would also interact with the screening guidelines recommended by JEMDSA guidelines and OSSA and could become part of a ‘diabetes care package’.^15,25^

The images were saved on the computer and at future eye screening, the current and previous images can be compared to track progression of diabetic retinopathy. Over 90% of the fundus and macula images obtained were of sufficient quality to be graded by the ophthalmology registrar. Patients were shown their eye images by the main author and educated on the importance of glucose control and why diabetes causes visual impairment. Patients from different backgrounds and educational levels understood visual impairment better when an image with their diabetic retinopathy was compared to an image where there was no diabetic retinopathy. Patients who had no evidence of diabetic retinopathy and who had a visual acuity of 6/12 or better were also educated with their own retinal images contributing to patients’ insights on why diabetes control is important.

The study in Cape Town found proliferative diabetic retinopathy in 6.1% and maculopathy in 15.2% of participants.^12^ In our study, the prevalence of proliferative diabetic retinopathy was lower at 2.7% or five patients, and the prevalence of observable maculopathy was 10.0% or 15 patients.

The WHO highlights that more than 80% of worldwide visual impairment is treatable and preventable, but because of the deficiency in eye care, delivery and provision, patients are still at risk of visual loss.^2^ Early detection of diabetes-related eye disease by using visual acuity screening and retinal examination whether with direct fundoscopy, indirect fundoscopy or fundus photography and the implementation of the referral guidelines into a primary healthcare clinic, can improve the early detection of preventable or treatable complications.

### Strengths and limitations

Potential limitations included the examination skills of the main author to screen for diabetic retinopathy and other ocular pathology. This was addressed by correlating the findings with the digital fundus images stored on a computer and discussing the findings with a registrar from the Department of Ophthalmology. The responses to the questions were accepted at face value and not validated. There may have been language barriers. Convenience sampling was used in the study as only known diabetic patients were included in the sample and some attending the Outpatient Department may have been missed.

## Conclusion

The study demonstrated that the Outpatient Department at NDH is falling short of the current recommended annual diabetic retinopathy screening protocol as recommended by OSSA and SEMDSA. Another concern is that almost 80% of patients had never been referred to an ophthalmologist for diabetic retinopathy screening.

The study found that the prevalence of eye pathology was high in this group of diabetic patients, although the prevalence of diabetic retinopathy was low compared to other studies from other South African regions.

This study has confirmed that it is possible and expedient to bring eye screening services to primary healthcare diabetic patients, where incident eye pathology can be identified and referred earlier.

### Recommendations

We recommend increasing the awareness of doctors and nursing staff attending to outpatients to enquire and examine diabetic patients for visual complaints. National or local initiatives of health promotion activities to increase patient awareness that uncontrolled diabetes can lead to visual impairment can be conducted by any healthcare worker. There is a need to educate both the general public and healthcare professionals about eye diseases and preventable or treatable conditions such as glaucoma, diabetic eye disease, age-related macular degeneration and genetic eye conditions.

The authors recommend the use of non-mydriatic fundus camera during eye screening of all diabetic clinic patients as part of routine care. A collaborative practice model with Optometry and Ophthalmology Departments could assist with the screening. We also suggest that within each healthcare district, at a primary level, a trained ophthalmic nurse/medical officer be placed and provided with a Snellen chart, a direct ophthalmoscope, an easy-to-operate tonometer and a non-mydriatic fundus camera to screen patients effectively.
